# Novel Mutations of *ABCB6* Associated with Autosomal Dominant Dyschromatosis Universalis Hereditaria

**DOI:** 10.1371/journal.pone.0079808

**Published:** 2013-11-05

**Authors:** Ying-Xia Cui, Xin-Yi Xia, Yang Zhou, Lin Gao, Xue-Jun Shang, Tong Ni, Wei-Ping Wang, Xiao-Buo Fan, Hong-Lin Yin, Shao-Jun Jiang, Bing Yao, Yu-An Hu, Gang Wang, Xiao-Jun Li

**Affiliations:** 1 Institute of Laboratory Medicine, Jinling Hospital, Nanjing University School of Medicine, Nanjing, PR China; 2 Department of Dermatology, Xijing Hospital, Fourth Military Medical University, Xi'an, PR China; 3 Department of Dermatology, Jinling Hospital, Nanjing University School of Medicine, Nanjing, PR China; 4 Department of Pathology, Jinling Hospital, Nanjing University School of Medicine, Nanjing, PR China; Charité Universitätsmedizin Berlin, NeuroCure Clinical Research Center, Germany

## Abstract

**Objective:**

Dyschromatosis universalis hereditaria (DUH) is a rare heterogeneous pigmentary genodermatosis, which was first described in 1933. The genetic cause has recently been discovered by the discovery of mutations in *ABCB6*. Here we investigated a Chinese family with typical features of autosomal dominant DUH and 3 unrelated patients with sporadic DUH.

**Methods:**

Skin tissues were obtained from the proband, of this family and the 3 sporadic patients. Histopathological examination and immunohistochemical analysis of ABCB6 were performed. Peripheral blood DNA samples were obtained from 21 affected, 14 unaffected, 11 spouses in the family and the 3 sporadic patients. A genome-wide linkage scan for the family was carried out to localize the causative gene. Exome sequencing was performed from 3 affected and 1 unaffected in the family. Sanger sequencing of *ABCB6* was further used to identify the causative gene for all samples obtained from available family members, the 3 sporadic patients and a panel of 455 ethnically-matched normal Chinese individuals.

**Results:**

Histopathological analysis showed melanocytes in normal control’s skin tissue and the hyperpigmented area contained more melanized, mature melanosomes than those within the hypopigmented areas. Empty immature melanosomes were found in the hypopigmented melanocytes. Parametric multipoint linkage analysis produced a HLOD score of 4.68, with markers on chromosome 2q35-q37.2. A missense mutation (c.1663 C>A, p.Gln555Lys) in *ABCB6* was identified in this family by exome and Sanger sequencing. The mutation perfectly cosegregated with the skin phenotype. An additional mutation (g.776 delC, c.459 delC) in *ABCB6* was found in an unrelated sporadic patient. No mutation in *ABCB6* was discovered in the other two sporadic patients. Neither of the two mutations was present in the 455 controls. Melanocytes showed positive immunoreactivity to ABCB6.

**Conclusion:**

Our data add new variants to the repertoire of ABCB6 mutations with DUH.

## Introduction

Dyschromatosis universalis hereditaria (DUH) is a group of heterogeneous pigmentary genodermatosis characterized by asymptomatic hypo- and hyper-pigmented macules of irregular size and shape which appear early in life; in fact, DUH most often manifests within the first year of life. DUH was first described in 1933 by Ichikawa and Higari [1] and was most commonly reported in Japan [2], but it was also reported in other Asian countries, Europe, South America, and Africa [3-10]. DUH was classified into two subtypes, DUH 1 (OMIM, 127500), an autosomal dominant disease, and DUH 2 (OMIM, 612715) an autosomal recessive disease. DUH with autosomal dominant transmission is generally reported. The locus for autosomal dominant DUH has been reported at chromosome 6q24-q25.2, between D6S1703 and D6S1708, spanning 10.2.Mbp [11]. Recently, Zhang and colleagues have reported that *ABCB6* mutations are responsible for DUH [12]. To further expand mutational spectrum of *ABCB6* gene, we investigated a Chinese family with typical features of autosomal dominant form of DUH and 3 unrelated patients with sporadic DUH, and found two novel mutations in *ABCB6*.

## Materials and Methods

### Ethics statement

The Ethics Committee of Jinling Hospital and Xijing Hospital approved the protocols used. The research adhered to the tenets of The Declaration of Helsinki. All participants gave written informed consent to participate in the study.

### Participant and Clinical data

**Figure 1:**
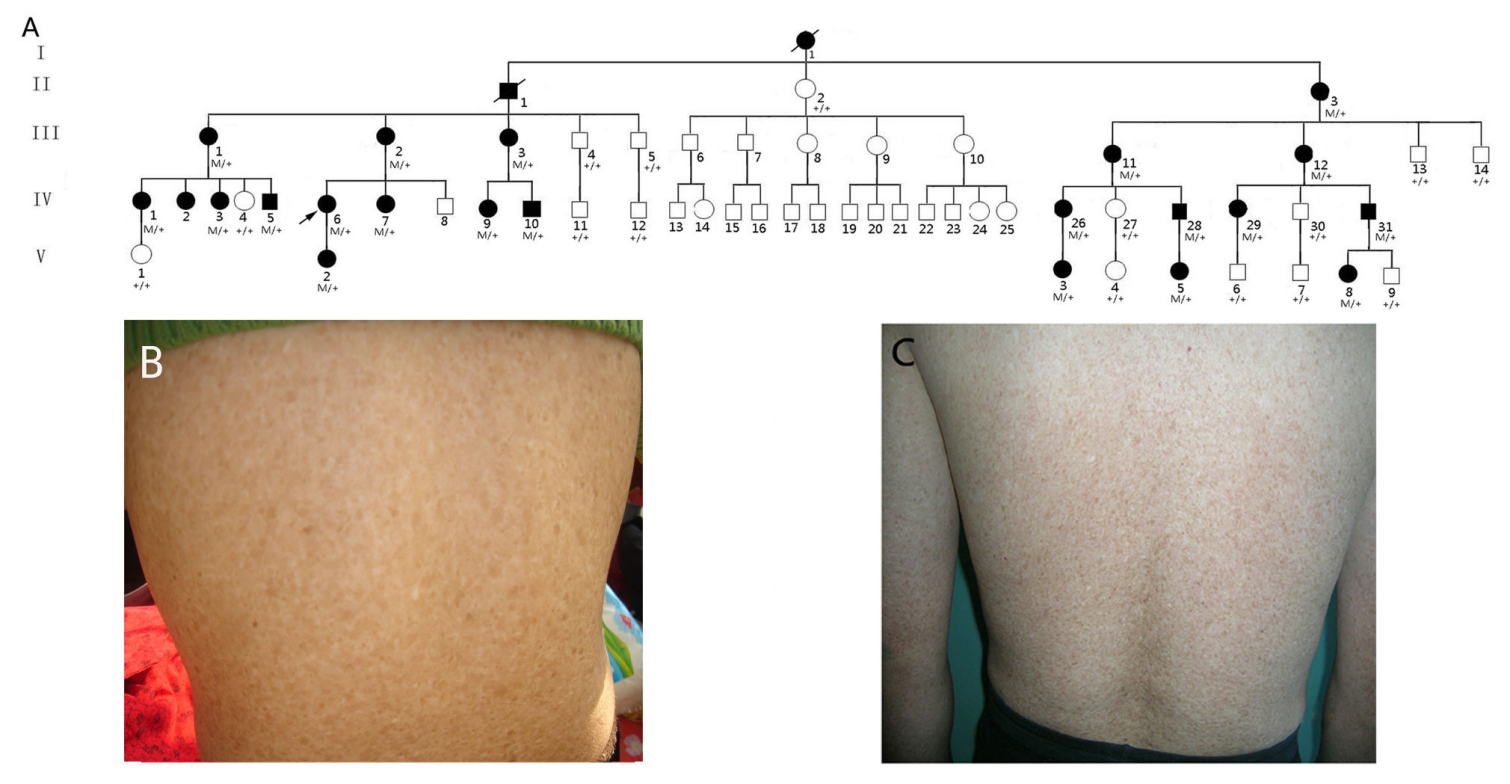
Family pedigree and skin phenotypes. (A) Family pedigree. M, mutation; +, normal. An arrow indicates the proband, IV-6. (B) Skin feature of the proband (IV-6) in the family with c.1663C > A. (C) Skin feature of an unrelated patient with sporadic DUH with the c.459 delC mutation.

The pedigree of a four-generation Han family with typical features of DUH is from the rural area of Shandong Province in China. The family showed an autosomal dominant inheritance pattern (Figure 1A) and was referred by Jinling Hospital, Nanjing University School of Medicine. The 3 unrelated patients with sporadic DUH diagnosed by Xijing Hospital, Fourth Military Medical University were also recruited. A complete family history was obtained, and 21 affected individuals (4 males and 17 females) were identified. Forty-six family members (21 affected, 14 unaffected and 11 spouses) in the family and the 3 unrelated patients with sporadic DUH participated in the study. Peripheral blood DNA samples were obtained from all available family members and the 3 sporadic patients with DUH. Careful physical examination revealed that all affected individuals in this family had a pigmentary disorder without the association of any other systemic disease. For example, the proband, Ⅳ-6, is a 33-year-old female with cutaneous hypo- and hyper-pigmented macules, 3–7 mm diameter, scattered over the entire body including the hands, feet, back, face, and scalp (Figure 1B). The skin at palms and soles, mucus membranes, teeth, nails, and hairs appeared normal. The hyperpigmented lesions were dark brown in color, while the hypopigmented lesions were light brown-to-white. The proband stated that the skin macules emerged within 2 months after birth and became darker when exposed to the sun, but there were no associated abnormal sensations. None of the affected members in this family was found to have skin cancer or ocular coloboma. The proband and the sporadic patient with the c.459 delC mutation provided written informed consent, as outlined in the PLOS consent form, to the publication of their photographs. Skin tissue samples were obtained from the proband and the 3 sporadic patients with DUH, as well as an age-gender matched normal control donated by a 35-year-old healthy Han Chinese female from cosmetic surgery. 

### Histopathological examination for melanocytes in skin tissues

Punch biopsies from the proband, the 3 sporadic patients and the normal control were taken, and each sample was prepared for light and electron microscopic investigation. For light microscopic analysis, 4-μm-thick histological sections were stained with methylene blue. For electron microscopic analysis, ultrathin sections were treated with methanolic uranyl acetate and lead citrate.

### Genome-wide genotyping for linkage analysis

A genome-wide linkage scan in the family was carried out to determine the chromosomal regions linked to DUH. Twenty family members (10 affected, 5 unaffected and 5 spouses) participated in this study. DNA samples were genotyped on the Affymetrix GeneChip Human Mapping 500K Array containing more than 500,000 SNPs. Array experiments were carried out according to the manufacturer’s protocol. The Affymetrix GeneChip Operating Software (GCOS) was used for image processing. Genotypes were called with the Affymetrix Genotyping Console Software (GTC 4.0). Parametric multipoint linkage analysis was performed with Merlin software under the assumption of autosomal-dominant inheritance with 99% penetrance, a disease allele frequency of 0.1%, and equal SNP allele frequency (50%).

### Exome-wide DNA capture and next-generation sequencing

Three µg qualified genomic DNA samples from each of the 4 members (3 affected, 1 unaffected) in the family were randomly fragmented by Covaris. The DNA with adapters on both ends was then amplified, purified, and hybridized to the Nimblegen SeqCap EZ Library (v2.0; http://www.nimblegen.com) for enrichment; high-throughput sequencing was performed for each captured library. Raw image files were processed by Illumina basecalling Software 1.7 for base-calling with default parameters and the sequences of each individual were generated as 90bp pair-end reads. 

### Detection of *ABCB6* gene and alignment analysis

*ABCB6* (NG_032110.1) coding regions and their intronic flanking sequences were amplified by PCR from genomic DNA and the products were then sequenced. DNA samples were obtained from the 21 affected, 14 unaffected family members and the 3 unrelated individuals with sporadic DUH. PCR primers targeting the 19 exons of *ABCB6* were designed using Exon-Primer from the UCSC Genome Browser. Furthermore, DNA samples from the 455 unrelated normal healthy Han Chinese individuals were amplified and sequenced to exclude SNP. ClustalW (version 1.83) was used to compare ABCB6 (NP_005680.1) with orthologs of *P.troglodytes* (XP_001161097.1), *M.mulatta*(XP_002799099.1), *F.catus* (XP_003991217.1), *M.musculus* (NP_076221.1), *C.elegants* (NP_001022812.1), *D.melanogaster* (NP_650503.1).

### Immunohistochemistry staining of ABCB6 for skin tissues

The affected skin tissues from the proband with the c. 1663 C>A mutation, the sporadic patient with the c.459 delC mutation and the normal control were fixed in 10% formalin and embedded in paraffin. Sections of 3μm were submitted to immunostaining with the monoclonal antibody against ABCB6 (HZ817454, Enzyme Chain Biotechnology Co., Ltd, Shanghai, China, dilution 1:400). The sections were dewaxed and subjected to antigen retrieval (pressure cooking for 1 minute at full pressure, 15psi, in 0.001M EDTA butter, pH 8.0). Immunohistochemistry staining of ABCB6 was processed by Dako Envision System (K5007, Dako, Danmark).

## Results

### Observation of melanocytes by light and electron microscope

**Figure 2:**
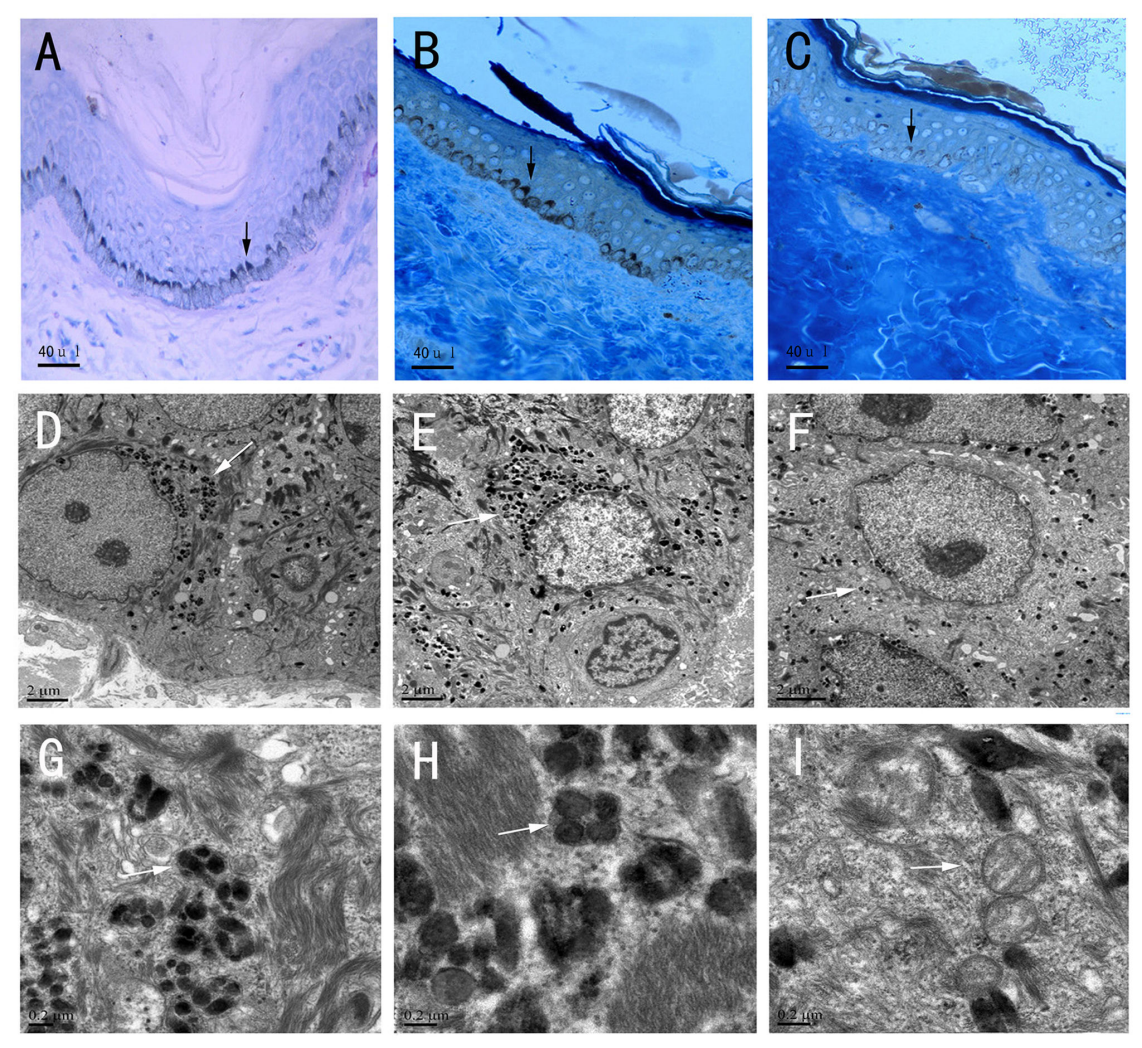
Skin histological examination under light and electron microscopy. (A-C) Sections of skin cutaneous tissues by methylene blue staining from the normal control, from the proband’s hyperpigmented area, from the proband’s hypopigmented area observed under light microscopy. (D-F) A melanocyte from the normal control’ skin tissue, from the proband’s hyperpigmented and from hypopigmented skin tissues observed by electron microscopy. (G-I) Melanosomes in the normal control’ melanocyte, in proband’s hyperpigmented and in hypopigmented melanocytes observed by electron microscopy. (B-C) Methylene blue staining of cutaneous tissues shows normal numbers of morphologically-intact melanocytes present in the basal layer of hypo- and hyper-pigmented skin areas. The content and distribution of mature melanosomes in hyperpigmented macules(E) were similar to as in the normal control(D), whereas the content in hypo-pigmented macule(F) was less than in the normal control(D). (G-H) Mature melanosomes in a melanocyte from the normal control’ tissue, from the proband’s hyperpigmented area. (I) Empty immature melanosomes in the stage II in a hypopigmented melanocyte. Arrows indicate melanocytes or melanosomes.

A normal number of morphologically-intact melanocytes were present in the basal layer in hypo- and hyper-pigmented skin areas from the proband (Figure 2B-C). Histopathologic analysis showed melanocytes in normal control’s skin tissue (Figure 2A, D, G) and the hyperpigmented areas from the proband (Figure 2B, E, H) contained more melanin or melanized, mature melanosomes than those within the hypopigmented areas (Figure 2C, F, I). Empty immature melanosomes were found in the hypopigmented melanocytes (Figure 2I). Comparing the content and distribution of mature melanosomes in melanocytes from the proband with those from the normal control, we found the content and distribution in hyperpigmented macule (Figure 2E) were similar to normal control (Figure 2D), whereas the content in hypo-pigmented macule (Figure 2F) was less than control (Figure 2D).

### Linkage analysis

Parametric multipoint linkage analysis of the family revealed two genetic linkage regions on chromosomes 2q35- q37.2 and 6p22. The former genetic linkage region spanned approximately 17Mbp with a HLOD score of 4.68 and the latter spanned nearly 300kbp with HLOD score of 4.59; no annotated gene was identified in 6p22. No significant linkage with markers on other chromosomal regions was identified in this DUH family (Figure S1).

### Exome sequencing

An average of 6.50 Gb bases per member was generated by exome sequencing. Approximately 3.1Gb bases were mapped to target exome region with a mean depth of 70.00-fold and nearly 99.28% of the targeted bases were covered sufficiently to pass our thresholds for calling SNPs, short insertions or deletions. The rate of nucleotide mismatch was below 0.3%. After filtering against exome databases (dbSNP135, 1000 Genome Project, YH database and HapMap project) by sequencing data analysis, 56 variants (39 SNPs and 17 deletions or insertions) in the three affected individuals were still remained (Table S1), but not existed in the unaffected control. Combined information from linkage analysis with exome sequencing, four genes (*USP40, ABCB6, SLC11A1* and *NCL*) with novel heterozygous mutations were selected as candidate genes in this family and *USP40, SLC11A1* and *NCL* were further excluded by Sanger sequencing. 

### Mutation validation and alignment of ABCB6

**Figure 3:**
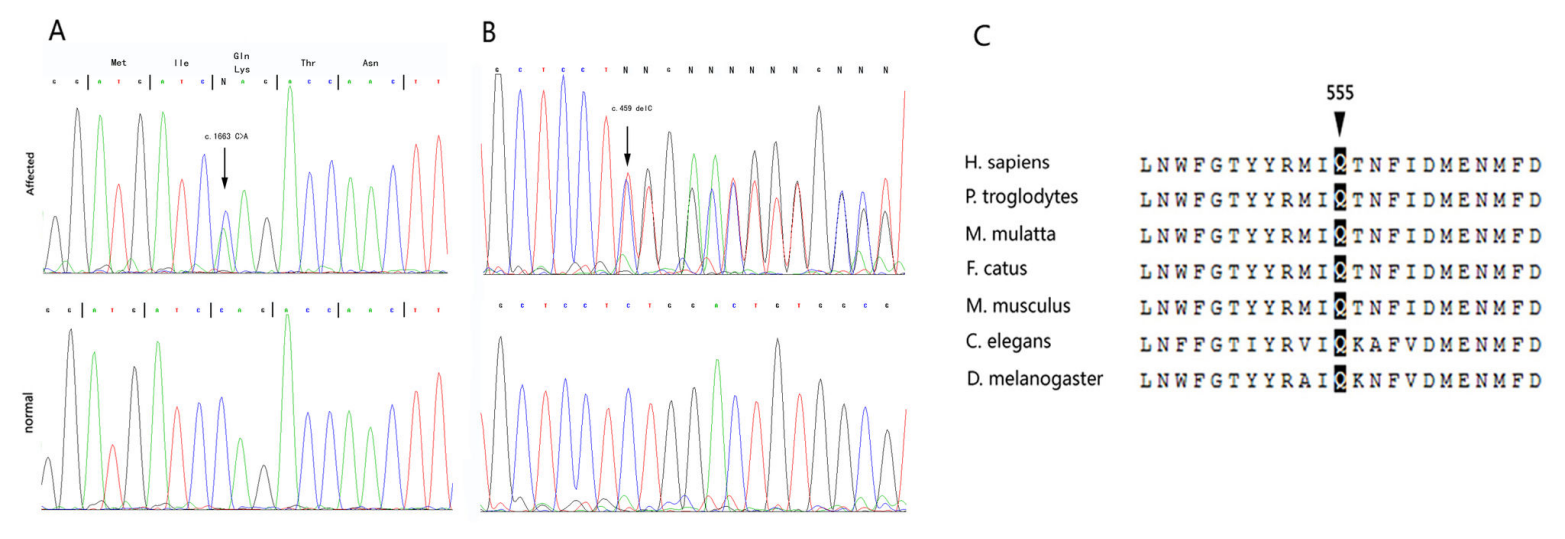
ABCB6 heterozygous mutations and sequence alignments. (A) Heterozygous mutations of c. 1663 C>A in the proband and (B) c.459 delC in a sporadic DUH patient. (C) A partial sequence of ABCB6. Arrows indicate the location of the mutations.

A missense mutation g. 5496 C>A, c.1663C > A, p. Gln555Lys in exon 11 of *ABCB6* (Figure 3A) was further identified in the 21 affected members, but absent in the 14 unaffected members from the family and completely cosegregated with the skin phenotype. An additional mutation in exon 1 of *ABCB6* (g.776 delC, c.459 delC) was detected (Figure 3B) in an unrelated sporadic patient with typical DUH (Figure 1C), however, no mutation in *ABCB6* was found in the other two patients. Mutations of c.1663 C>A and c.459 delC of in *ABCB6* were not listed in the NCBI SNP database (dbSNP). Furthermore, sequencing was utilized in the 455 ethnically-matched normal Chinese individuals to exclude the mutations as SNPs. The mutation of p.Gln555 in ABCB6 is located in a conserved region of the protein (Figure 3C). 

### Immunohistochemistry of ABCB6

**Figure 4:**
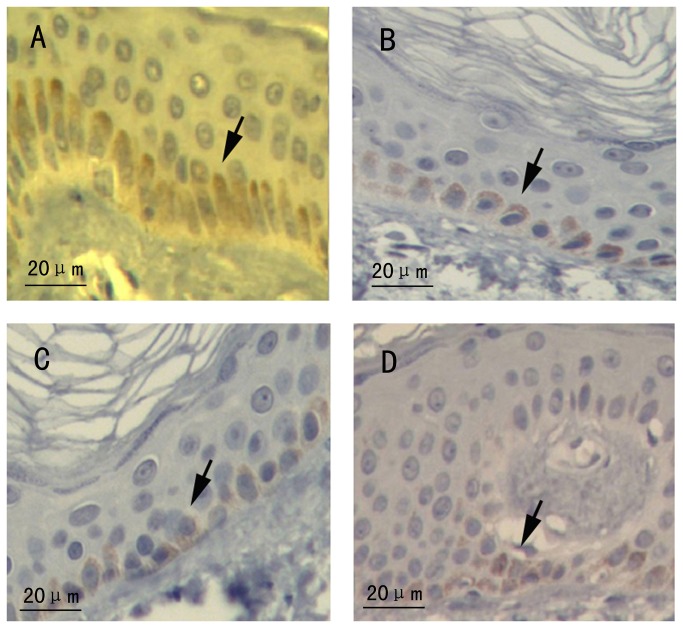
Immunohistochemistry staining of ABCB6 observed under light microscopy. (A) Section of skin tissue from the normal control. (B) Section of hyperpigmented tissue from the proband with c. 1663 C>A. (C) Section of hypopigmented area from the proband. (D) Section of skin tissue from the patient with c.459 delC. (A-D) All of the melanocytes were positive immunoreactivity for ABCB6. Arrows indicate melanocytes.

Immunohistochemistry analysis showed all of the melanocytes were positive immunoreactivity to ABCB6 (Figure 4A-D).

## Discussion

In the study, we found a c. 1663 C>A, p.Gln555Lys heterozygous mutation of *ABCB6* in exon 11, which perfectly cosegregated with the disorder in the family. Alignment comparative analysis showed that Gln555 was highly conserved (Figure 3C), which implied that the residue was key to normal biological function. A deleted mutation in exon 1 (c.776 delC) of *ABCB6* was further confirmed in an unrelated Chinese patient with sporadic DUH. The loci of c.1663CA and c.459 delC of *ABCB6* as SNPs were excluded in a panel of 455 unrelated healthy Chinese individuals. Combining these data, we suggest that the mutations c.1663CA and c.459 delC of *ABCB6* be the genetic cause of the disease in the patients with the familial and the sporadic DUH. 

Human *ABCB6* (OMIM 605452) was cloned in 2000 [13] and is located on chromosome 2q36. *ABCB6* contains 19 exons in the protein-coding region and belongs to the ABC transporter family. ABCB6 is involved in the active transport of peptides, steroids, polysaccharides, amino acids, phospholipids, ions, bile acids, and pharmaceutical drugs[13]. *ABCB6* is widely expressed in tissues [13-17] with high expression in heart, skeletal muscles, fetal liver, melanocytes and melanoma cells [13,15,18]. Previous studies have indicated that ABCB6 is localized in the outer mitochondrial membrane [18] and was proposed to serve as a mammalian mitochondrial porphyrin transporter. Recent studies have revealed that ABCB6 is glycosylated in multiple cell types and it is localized in the endoplasmic reticulum, Golgi apparatus, lysosome, and plasma membrane, rather than in the mitochondria [19,20]. Skin melanin is normally synthesized by melanocytes located in the basal layer of the epidermis. Melanin synthesis takes places in intracellular lysosome-related organelles termed melanosomes derived from the endoplasmic reticulum, which bud off the Golgi complex and are extruded into the surrounding keratinocytes [21]. Specialized melanocytic enzymes and structural proteins are trafficked and assembled into the melanosomal particle during a maturation process from an empty vacuole to a striated melanin filled organelle, designated in four stages I–IV. In the study, we found a normal number of melanocytes in the basal layer in both hyper- and hypo-pigmented areas, however, the amount of mature melanosomes in normal control and hyperpigmented skin areas was considerably higher than in hypopigmented area. Additionally, many immature melanosomes were observed in hypopigmented skin region. These findings suggested that DUH might be a disorder of melanosome maturing,, rather than a disorder of melanocyte number. ABCB6, as a multifunctional transporter protein, is highly expressed in melanocytes, suggesting that it might participate in the transport of specialized enzymes and proteins, which are necessary for melanin synthesis. Therefore, concluding from the phenotype of a defect of melanosome maturing in the hypopigmented macule, we hypothesize the mutation of c.459 delC to be hypomorphic, because it resulted in a frameshift within the coding sequence and brought about premature translation termination codon and might further lead to nonsense-mediated mRNA decay and haploinsufficiency [22]. As for the heterozygous missense mutation of c. 1663 C>A, we also suggested it to be hypomorphic mutation, because the similar skin phenotype was found in the patient with c. 1663 C>A. 

Previous studies revealed mutations in *ABCB6* were associated with at least four Mendelian phenotypes: homozygous or compound heterozygous mutations of *ABCB6* were associated with the negative Lan blood type [16]. In particular, individuals with the negative Lan blood type did not exhibit any clinical consequences, suggesting that the gene was dispensable for erythropoiesis in humans. Heterozygous missense mutations of *ABCB6* (p.R375Q and p.R375W) were found in dominant familial pseudohyperkalemia, which were likely to be gain-of-function mutations suggested by Andolfo et al.[17]. Heterozygous missense mutations (p.L811V and p.A57T) in *ABCB6* caused iris coloboma, aniridia, chorioretinal coloboma [14]. The phenotypes might be explained by haploinsufficiency. Mutations of p.S170G, p.L356P, p.G579E in *ABCB6* resulted in DUH [12]. We found novel heterozygous mutations (c. 1663 C>A and c.459 delC) in *ABCB6* with autosomal dominant DUH, and undoubtedly, our data expand the mutational spectrum of *ABCB6*. Intriguingly, ABCB6 is widely expressed in tissues, but the mutations of c. 1663 C>A and c.459 delC in *ABCB6* are only associated with skin dyschromatosis, presumably because the function of ABCB6 can be compensated from elsewhere in other organs. Furthermore, heterozygous mutations at different positions in the *ABCB6* gene resulted in completely different phenotypes. The cause is still unclear, and a potential explanation might be due to the diversity of the involved domains or interacting molecules [23]. However, the underlying pathomechanism of the pigmentary changes in autosomal-dominant DUH is unclear. Functions of the ABCB6 transporter will require further studies, including disease-specific mutations associated with specific organ phenotypes. 

## Supporting Information

Figure S1 **Parametric multipoint linkage analysis in the family with DUH.** Two genetic linkage regions on chromosomes 2q35- q 37.2 and 6p22 with HLOD scores 4.68 and 4.59, respectively.
(TIF)

Table S1 **Sequence variants found by exome sequencing.**
(DOC)
