# Supplementary figures and images for: Novel Mutations of ABCB6 Associated with Autosomal Dominant Dyschromatosis Universalis Hereditaria
Source: PLoS One. 2013 Nov 5;8(11):e79808. doi: 10.1371/journal.pone.0079808 (PMC3818219; doi:10.1371/journal.pone.0079808)

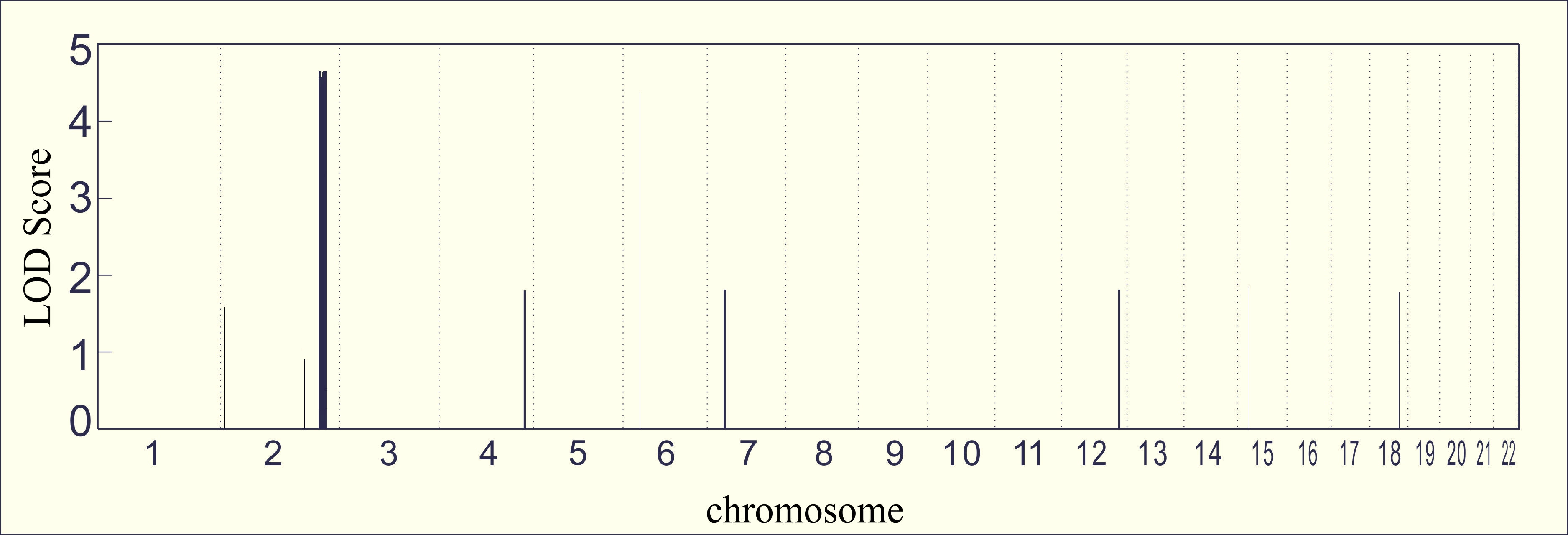

Supplement: Figure S1 — Parametric multipoint linkage analysis in the family with DUH. Two genetic linkage regions on chromosomes 2q35- q 37.2 and 6p22 with HLOD scores 4.68 and 4.59, respectively. (TIF) [file pone.0079808.s001.tif]
