# Supplementary material for: Novel Mutations of ABCB6 Associated with Autosomal Dominant Dyschromatosis Universalis Hereditaria
Source: PLoS One. 2013 Nov 5;8(11):e79808. doi: 10.1371/journal.pone.0079808 (PMC3818219; doi:10.1371/journal.pone.0079808)
Supplement: Table S1 — Sequence variants found by exome sequencing. (DOC) [file pone.0079808.s002.doc]

**Table S1. Sequence variants found by exome sequencing**

| chromosome | chromosome position | gene | transcript ID | exon/intron | variant | amino acid change | notation |
| --- | --- | --- | --- | --- | --- | --- | --- |
| 1 | 117146563 | IGSF3 | CCDS30813.1 | exon 5 | c.C1307T | p.T436M | missence |
| 2 | 27535310 | MPV17 | ENST00000489478 | exon 5 | n.482G>A |  | ncRNA |
| 2 | 97910960 | ANKRD36 | CCDS54379.1 | exon 71 | c.A4636T | p.R1546W | missense |
| 2 | 98127647 | ANKRD36B | NM_025190 | exon 39 | c.C3674T | p.S1225L | missense |
| 2 | 219247744 | SLC11A1 | CCDS2415.1 | exon 2 | c.C69G | p.S23R | missense |
| 2 | 220078217 | ABCB6 | CCDS2436.1 | exon 11 | c.C1663A | p.Q555K | missense |
| 2 | 232325426 | NCL | CCDS33397.1 | exon 4 | c.T765A | p.E254D | missense |
| 2 | 232325429 | NCL | CCDS33397.2 | exon 4 | c.A762T | p.D255E | missense |
| 2 | 234434150 | USP40 | CCDS46547.1 | exon 13 | c.G1817A | p.G606E | missense |
| 3 | 49723739 | MST1 | CCDS33757.2 | intron 8 | c.1016+7T>G |  | doner site |
| 4 | 144922436 | GYPB | CCDS54809.1 | exon 2 | c.A38C | p.E13A | missense |
| 6 | 32497896 | HLA-DRB5 | CCDS4751.1 | intron 1 | c.1000+6T>A |  | doner site |
| 6 | 161519381 | MAP3K4 | CCDS34565.1 | exon 17 | c.C3596T | p.A1199V | missense |
| 7 | 99795240 | STAG3 | ENST00000440830 | intron 1 | n.140+4A>T | - | ncRNA |
| 7 | 100639177 | MUC12 | CCDS55139.1 | exon 2 | c.C5333T | p.S1778L | missense |
| 8 | 12041190 | FAM86B1 | ENST00000321602 | exon 4 | c.G376A | p.G126R | missense |
| 8 | 12044201 | FAM86B1 | NM_001083537 | intron 4 | c.374+8T>C |  | doner site |
| 8 | 17915126 | ASAH1 | CCDS6005.1 | exon 14 | c.G1153A | p.V385I | missense |
| 9 | 90749760 | FAM75C2 | NM_001166137 | exon 1 | c.T112C | p.F38L | missense |
| 9 | 97087823 | FAM22F | CCDS47994.1 | exon 2 | c.C410G | p.S137C | missense |
| 9 | 97087857 | FAM22F | CCDS47994.1 | exon 2 | c.C376T | p.L126F | missense |
| 10 | 71969322 | PPA1 | CCDS7299.1 | exon 7 | c.A631G | p.K211E | missense |
| 10 | 135440080 | FRG2B | CCDS44502.1 | exon 1 | c.T167C | p.I56T | missense |
| 10 | 135440222 | FRG2B | CCDS44502.1 | exon 1 | c.G25A | p.D9N | missense |
| 11 | 1017529 | MUC6 | CCDS44513.1 | exon 31 | c.C5272T | p.H1758Y | missense |
| 12 | 9590052 | DDX12 | NR_033399 | exon 4 | n.622C>T |  | ncRNA |
| 12 | 50746164 | FAM186A | CCDS44878.1 | exon 4 | c.T4451C | p.I1484T | missense |
| 12 | 50746243 | FAM186A | CCDS44878.1 | exon 4 | c.A4372C | p.T1458P | missense |
| 12 | 50829263 | LARP4 | CCDS41782.1 | intron 5 | c.399-8T>C |  | acceptor site |
| 14 | 19378020 | OR11H12 | CCDS32017.1 | exon 1 | c.C427A | p.R143S | missense |
| 15 | 28483903 | HERC2 | CCDS10021.1 | exon 23 | c.G3593T | p.G1198V | missense |
| 15 | 34678911 | GOLGA8A | CCDS10038.1 | exon 3 | c.G200C | p.R67P | missense |
| 16 | 1279438 | TPSB2 | NM_024164 | exon 4 | c.G253A | p.A85T | missense |
| 17 | 15343524 | FAM18B2-CDRT4 | NM_001204478 | intron 6 | c.*42+1G>A |  | doner site |
| 17 | 17719515 | SREBF1 | CCDS11189.1 | intron 11 | c.2214+6T>G |  | doner site |
| 17 | 45234327 | CDC27 | CCDS11509.1 | exon 7 | c.G794A | p.G265D | missense |
| 18 | 14542619 | POTEC | CCDS45835.1 | intron 1 | c.521+6T>C |  | doner site |
| 19 | 53856776 | ZNF845 | CCDS46170.1 | exon 3 | c.A2848G | p.K950E | missense |
| 22 | 16449617 | OR11H1 | CCDS33594.1 | exon 1 | c.T188C | p.V63A | missense |
| 1 | 89851100 | GBP6 | NM_198460 | 3’-UTR | c.*72_*73insT |  | 3’-UTR |
| 2 | 198355135 | HSPD1 | ENST00000491249 | exon 1 | n.161_162insCA |  | ncRNA |
| 2 | 202344180 | STRADB | ENST00000466770 | exon 1 | n.316delT |  | ncRNA |
| 4 | 39501723 | UGDH | NM_003359 | 3’-UTR | c.*40delT |  | 3’-UTR |
| 6 | 4944004 | CDYL | ENST00000469671 | exon 4 | n.689_690insA |  | ncRNA |
| 6 | 57512794_57512796 | PRIM2 | NM_000947 | intron 14 | C.*86-3_*86-1delATT |  | donor site |
| 7 | 6037058 | PMS2 | CCDS5343.1 | intron 6 | c.708-4delT |  | acceptor site |
| 7 | 88424366_88424367 | C7orf62 | NM_152706 | intron 1 | c.-104-7_-104-6delTT |  | acceptor site |
| 9 | 95618606 | ZNF484 | CCDS35066.1 | intron 1 | c.16-6delT |  | acceptor site |
| 10 | 51623630 | RP11-481A12.5 | ENST00000431974 | exon 1 | n.214delA |  | ncRNA |
| 10 | 51623648 | RP11-481A12.5 | ENST00000431974 | exon 1 | n.232delG |  | ncRNA |
| 10 | 63977012_63977016 | RTKN2 | CCDS7263.1 | intron 8 | c.889-8_889-4delTTTTT |  | acceptor site |
| 12 | 6955865 | U47924.25 | ENST00000422785 | 3’-UTR | C.*86_*87insTGTG |  | 3’-UTR |
| 12 | 8693419 | CLEC4E | NM_014358 | 5’-UTR | c.-27_-26insGAGA |  | 5’-UTR |
| 17 | 75212479_75212491 | SEC14L1 | NM_001143999 | 3’-UTR | c.*2374_*2386delGTAGGTAGGGTTC |  | 3’-UTR |
| 22 | 43035850_43035851 | ATP5L2 | NM_001165877 | 3’-UTR | c.*128_*129delAA |  | 3’-UTR |
| X | 30877802_30877804 | TAB3 | NM_152787 | 5’-UTR | c.-90-9_-90-7delTTT |  | 5’-UTR |
